# The impact of emotional support on healthcare workers and students coping with COVID-19, and other SARS-CoV pandemics – a mixed-methods systematic review

**DOI:** 10.1186/s12913-023-09744-6

**Published:** 2023-07-13

**Authors:** Marja Härkänen, Adriana López Pineda, Susanna Tella, Sanu Mahat, Massimiliano Panella, Matteo Ratti, Kris Vanhaecht, Reinhard Strametz, Irene Carrillo, Anne Marie Rafferty, Albert W. Wu, Veli-Jukka Anttila, José Joaquín Mira

**Affiliations:** 1 Department of Nursing Science, University of Eastern Finland, Yliopistoranta 1c, Kuopio, Finland; 2 Department of Clinical Medicine, Miguel Hernandez University, San Juan de Alicante, Spain; 3 Present Address: The Foundation for the Promotion of Health and Biomedical Research of Valencia Region, Alicante, Spain; 4 LAB University of Applied Sciences, Lappeenranta, Finland; 5 Department of Translational Medicine (DIMET), Università del Piemonte Orientale, Novara, Italy; 6 Leuven Institute for Healthcare Policy, Department of Public Health & Primary Care, University of Leuven, Leuven, Belgium; 7 Department of Quality Management, University Hospitals Leuven, Leuven, Belgium; 8 Wiesbaden Business School of RheinMain University of Applied Sciences, Wiesbaden, Germany; 9 Health Psychology Department, Miguel Hernández University, Elche, Spain; 10 Florence Nightingale Faculty of Nursing, Midwifery & Palliative Care, King’s College, London, UK; 11 Johns Hopkins Bloomberg School of Public Health, Johns Hopkins School of Medicine, Baltimore, MD USA; 12 Helsinki University Hospital, Helsinki, Finland

**Keywords:** COVID-19, SARS, Resilience, Healthcare, Emotional, Support, Second victim, Systematic review

## Abstract

**Background:**

Pandemics such as COVID-19 pose threats to the physical safety of healthcare workers and students. They can have traumatic experiences affecting their personal and professional life. Increasing rates of burnout, substance abuse, depression, and suicide among healthcare workers have already been identified, thus making mental health and psychological wellbeing of the healthcare workers a major issue. The aim of this systematic review is to synthesize the characteristics of emotional support programs and interventions targeted to healthcare workers and students since the onset of COVID-19 and other SARS-CoV pandemics and to describe the effectiveness and experiences of these programs.

**Method:**

This was a mixed method systematic review. The Preferred Reporting Items for Systematic Reviews and Meta-Analyses (PRISMA) guidelines were followed, and the review was registered on PROSPERO [CRD42021262837]. Searches were conducted using Medline, CINAHL, PsycINFO, Cochrane Library, and Scopus databases. The COVIDENCE systematic review management system was used for data selection and extraction by two independent reviewers. The JBI (Joanna Briggs Institute) critical appraisal tools were used to assess the quality of selected studies by two additional reviewers. Finally, data extraction and narrative analysis were conducted.

**Results:**

The search retrieved 3161 results including 1061 duplicates. After screening, a total of 19 articles were included in this review. Participants in studies were nurses, physicians, other hospital staff, and undergraduate medical students mostly working on the front-line with COVID-19 patients. Publications included RCTs (*n* = 4), quasi-experimental studies (*n* = 2), cross-sectional studies (*n* = 6), qualitative interview studies (*n* = 3), and systematic reviews (*n* = 4). Most (63.4%) of the interventions used online or digital solutions. Interventions mostly showed good effectiveness (support-seeking, positive emotions, reduction of distress symptoms etc.) and acceptance and were experienced as helpful, but there were some conflicting results.

**Conclusion:**

Healthcare organizations have developed support strategies focusing on providing emotional support for these healthcare workers and students, but it is difficult to conclude whether one program offers distinct benefit compared to the others. More research is needed to evaluate the comparative effectiveness of emotional support interventions for health workers.

**Supplementary Information:**

The online version contains supplementary material available at 10.1186/s12913-023-09744-6.

## Background

Pandemics such as SARS, MERS, and COVID-19 pose threats to the physical safety of healthcare workers (HCWs), trainees and students. Frontline HCWs and students exposed to these pandemic viruses can have traumatic experiences affecting their personal and professional lives in the short and longer term. Working in healthcare institutions during a pandemic generates a significant emotional burden for HCWs [1]. Increasing rates of burnout, substance abuse, depression, and suicide among HCWs in many countries had already been identified prior to the COVID-19 pandemic, making mental health and psychological wellbeing of the HCWs a major issue [2, 3]. Unique demands posed by major pandemics have placed HCWs at an additional risk for mental health problems [4, 5]. Long working days and unpredictable courses of disease increase feelings of stress, helplessness, and fear [6–8].

Pandemic put HCWs at risk for depression, anxiety, post-traumatic stress disorder (PTSD) and moral injury [9–11]. Maunder and colleagues [12] found higher levels of anxiety and stress among HCWs during the 2003 SARS pandemic. Usually, these are related to workforce shortage and can lead to prolonged mental health problems. The widespread disruptions triggered by the COVID-19 pandemic increased attention to the mental health and wellbeing of HCWs. The unprecedented scale and duration of the crisis, social isolation, and unique professional demands have placed frontline HCWs at additional risk of developing mental health problems [13, 14]. Similarly, healthcare trainees working on the frontline were subjected to great psychological distress [15, 16]. In addition to personal suffering, this situation has jeopardized the HCWs ability to care for patients.

Several international agencies have suggested the need to provide support for the HCWs and trainees during and after pandemics. The World Health Organization (WHO) [17] suggested the need of psychological services such as directed psychological counselling and interventions to improve the emotional well-being of HCWs. Researchers have suggested several interventions such as providing social support, psychological services, adequate personal protection including vaccination and a safe work environment, financial support and incentives, and enhancing capabilities through continuous education and training [7, 18].

Healthcare organizations have developed support strategies focusing on providing emotional support for HCWs and students [19]. However, there are limited data on the efficacy and efficiency of these programs and interventions. There is also a lack of evidence regarding what kind of interventions and support programs are more helpful for HCWs and students working in high-risk environments such as pandemics [20]. It is also difficult to foresee if the well-intentioned interventions can decrease the psychological distress experienced by HCWs [21]. Furthermore, research has demonstrated that in the peak phase of crisis might, HCW might not give priority to psychological interventions and be reluctant to use the services offered to them [22].

Although there have been related systematic reviews, none correspond precisely to the aims, context, or timeframe of this review [23*, 24*, 25*, 26*]. Previous systematic reviews have aimed to study medical students support, interventions to reduce HCWs stress or mental health symptoms, or e-mental health interventions. There are also several published scoping or rapid reviews related to this topic. These found limitations to rapidly map recently developed brief interventions to meet the need for information raised by the COVID-19 pandemic to support frontline HCWs. There is also available information about experiences with recently implemented interventions.

This systematic review aims to synthesize the characteristics of emotional support programs and interventions targeted to HCWs and students since the onset of COVID-19 and other SARS-CoV pandemics. A mixed-methods approach [27] was employed to capture both the effectiveness of interventions and experiences with them from the perspective of HCWs and students.

## Methods

### Design

This is a mixed-methods systematic review including both quantitative and qualitative studies, as well as systematic reviews.

The Preferred Reporting Items for Systematic Reviews and Meta-Analyses (PRISMA) [28] guidelines were followed. This review was registered at PROSPERO—International prospective register of systematic reviews under the registration number CRD42021262837 [29].

The review question, based on the PICO method was the following:

- P = healthcare workers and students
- I = emotional support programs / interventions
- C = not applicable
- O = impact of these programs on mental health and well-being
  - What types of emotional support programs / interventions have healthcare professionals, trainees, and students accessed during and after COVID-19, SARS, MERS pandemics and what was the impact, defined as effectiveness and experiences, of these programs?

### Eligibility criteria

We included all research documents reporting support programs or interventions to improve the emotional well-being of HCWs. The inclusion criteria were: (a) peer-reviewed research articles: systematic reviews, quantitative, or qualitative studies, b) study participants: health professionals or students exposed to COVID-19, MERS or SARS pandemics in all health care and academic settings (no restriction on age, gender, years of experience, and/or healthcare profession), and c) participation in emotional support programs or interventions received following the COVID-19, MERS, or SARS pandemics. The exclusion criteria were: a) editorials, discussion papers, case studies, comments, letters, book chapters, and scoping or rapid reviews, b) published other language than English, Finnish, Spanish, German, Nepali, Indian, French, or Italian. c) no participation in the support program / intervention during the COVID-19, MERS, or SARS pandemics by HCWs, trainees or students, and d) no report of the impact (effectiveness and experiences) of the intervention or programs.

### Data search

Systematic searches were conducted by two authors (MH & SM) with the consultation of a medical librarian. Searches were made using following databases: Medline, Cumulative Index of Nursing and Allied Health Literature (CINAHL), PsycINFO, Cochrane Library, and Scopus for publications within years 2003–2021 to capture the most recent pandemics and epidemics (severe acute respiratory syndrome, SARS; influenza A - H1N1; and SARS-CoV-2). Only peer reviewed full-text articles from healthcare fields (nursing, medicine, and health education) were included. No geographical limitations were made for included studies. After many test searches, the final searches were conducted in 2nd of July 2021. Used search terms are described in Table 1.

**Table 1:** The used search terms

nurs*, midwife* physician*, “medical staff”, “healthcare practitioner*”, “healthcare professional*”, “health professional*”, “health practitioner*”, “health personnel”, “health care provider*”, “healthcare worker*”, “healthcare student*”, “nurse student”, “medical student”, “healthcare trainee”, “nurse trainee”, “medical trainee”
AND
“Second victim*”, experience*, feelings*, response*, psychological, emotion*, trauma*, “psychosomatic symptom*”, resilience*, “mental health”, well-being, “adaptative response”, “acute stress”, distress, stress*
AND
support* OR coping* OR psychological first aid*
AND
“COVID-19”, “SARS-CoV*”, “Coronavirus 2019*”, SARS*, MERS*, “MERS-CoV”, “coronavirus disease”
AND
impact, effect*, intervention*, implement*

### Study selection and data extraction

The COVIDENCE systematic review management system was used for data selection and extraction. Two reviewers (MH & SM) independently screened the titles and abstracts of all records retrieved after applying the established search strategy, and disagreements between the reviewers were resolved by discussion and mutual consent. Two reviewers (ST & AL) independently reviewed full-text documents and selected those which met the inclusion criteria. Each excluded study and the reason for its exclusion was recorded. Disagreement were resolved by as third reviewer (MH) or by discussion between reviewers. Then, one reviewer (MH) extracted data from the included studies using the COVIDENCE tool. Data extraction was conducted based on publication details (title, journal, author, year of publication), country of the study location, study design, virus pandemic, type of study participants, sample size (the number of included studies for reviews), aims, study intervention or support program and the findings on the impact (effectiveness and experiences) of the intervention or programs.

### Quality evaluation

Two reviewers (ST & AL) independently evaluated the risk of bias of the included studies using the JBI’s (Joanna Briggs Institute) critical appraisal tool [30]. Any potential discrepancies between reviewers were discussed and if required, a third reviewer was consulted. We considered that the methodological quality of the studies was poor when they meet less than 50% of items of the appraisal instrument (i.e. the response ‘Yes’ was given in less than 50% of the questions of the total tool questions).

### Data synthesis

Study characteristics were tabulated by the study design. A descriptive narrative synthesis of the studies was conducted comparing the type and content of the emotional support programs or interventions, and the impact (effectiveness and experiences) of these programs or interventions. Effectiveness refers to the relationship between an intervention and clinical or health outcomes. Feasibility (experience) refers to whether an intervention is practical, appropriateness is about how an intervention relates to the given context. Meaningfulness relates to the personal experience, opinions, values, thoughts, beliefs, and interpretations of participants [27]. Additionally, we reported the quality evaluation of the included studies. We emphasized those rated as poor quality to help to assess the evidence.

## Results

### Study selection

**Fig. 1:**
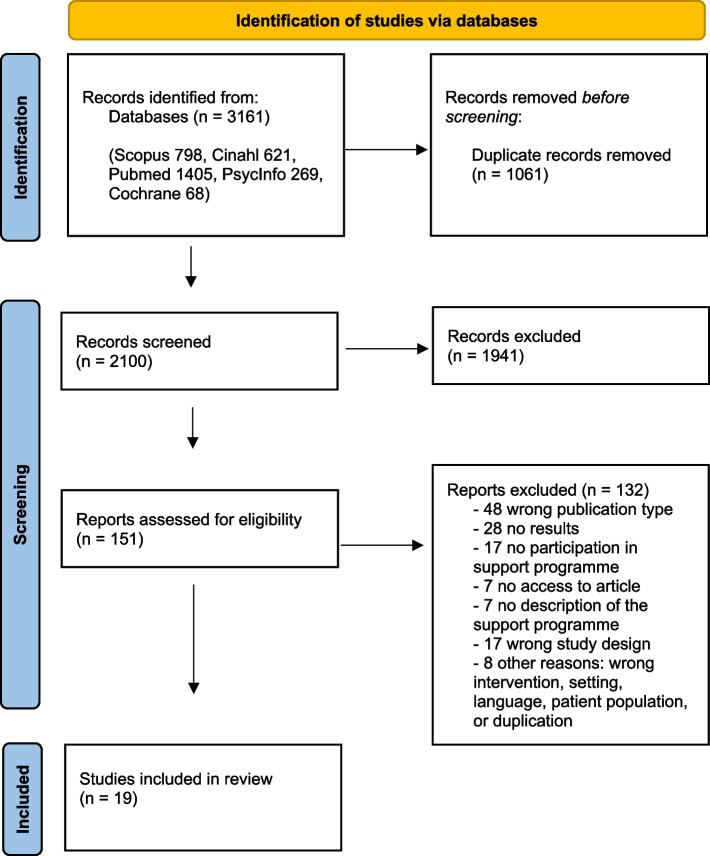
PRISMA flow diagram describing the study selection

The search retrieved 3161 results, which included 1061 duplicates. After removing duplicates, 2100 articles were screened, and 19 articles were included. The selection process is shown in the PRISMA flow diagram (Fig. 1).

### Quality evaluation

The results of the quality evaluation of studies are presented in Online Only materials 1, 2, 3, 4 and 5. The methodological quality of the RCT by Procaccia et al. [31*], the review by Buselli et al. [24*], Drissi et al. [25*] and the cross-sectional studies by Geoffroy et al. [32*] and Teall et al. [33*] were considered poor because of the high risk of bias in most indicators.

### Study characteristics

Included studies (*n* = 19) were conducted in USA (*n* = 5), UK (*n* = 4), Italy (*n* = 2), Spain (*n* = 1), Taiwan (*n* = 1), France (*n* = 1), Canada (*n* = 1), Singapore (*n* = 1), Iran (*n* = 1), Australia (*n* = 1), and one study (*n* = 1) where authors came from United Arab Emirates, Spain, and Morocco (Table 2). The language of all included studies was English.

**Table 2:** Emotional support programs and interventions (*n* = 19) for healthcare professionals, and students following pandemics

**Study**	**Participants**	**Emotional support programmes and interventions**
**Intervention studies (*n* = 6)**		
Amsalem et al. 2022 [34*], USA	Nurses **(*n* = 237)**, physicians **(*n* = 52)** and emergency medical technicians **(*n* = 30)**	The 3 min **video** to increase treatment-seeking by healthcare workers during the COVID-19 pandemic: it derived a 45 min interview of a 28-year-old intensive care unit female nurse, who described her difficulty in coping with her life stressors, how she faced her anxieties and depression, her prior false assumptions about treatment and how she overcame them.
Chen et al. 2006 [35*], Taiwan	Nursing staff **(*n* = 116)**	In-service training, **manpower allocation, gathering sufficient protective equipment, and establishment of a mental health team** were included.
Chochol et al. 2021 [36*], USA	Child and Adolescent Psychiatry (CAP) trainees **(*n* = 6)**	Eight 60-min sessions held every 2 weeks were co-facilitated by a psychologist and psychiatrist who developed the curricular content. Five of the eight semi-structured sessions combined a **brief emotional awareness enhancing module with a Balint-based approach** to case review.
Coifman et al. 2021 [37*], USA	Medical personnel, support staff and emergency responders **(*n* = 28)**	Low-dose or high-dose intervention, one time daily for 1 week via **smartphone application**. Each daily intervention included expressive writing, adaptive emotion regulation activity and (one vs two) positive emotion activities, lasting 3–6 min a day.
Fiol-DeRoque et al. 2021 [38*], Spain	Frontline health care workers** (*n* = 482)**	the PsyCovidApp intervention (a **mobile phone-based app** targeting emotional skills, healthy lifestyle behavior, burnout, and social support) or a control app (general recommendations about mental health care) for 2 weeks
Procaccia et al. 2021 [31*], Italy	Healthcare professionals **(*n* = 55)** in the frontline	**Expressive writing (EW) intervention**. EW is a tool through which subjects describe their deepest thoughts and feelings about emotional events.
**Cross-sectional survey studies (*n* = 6)**		
Blake et al. 2020 [39*], UK	Hospital employees **(*n* = 819)** from any site of an acute hospital	**Supported Wellbeing Centres**: The intention was that the centres would be relaxing spaces, and as such they had comfortable seating, relaxing music, low-level lighting, plants and an aromatherapy pod. Refreshments were also available.
Geoffroy et al. 2020 [32*], France	Hospital workers **(*n* = 149)**	The **psychological assistance hotline** team was composed of certified psychologist volunteers.
Monette et al. 2020 [40*], USA	Emergency clinicians **(*n* = 68)**	a **video-based debriefing program** (Zoom) to support emergency clinician well-being
Petrella et al. 2021 [41*], UK	All staff working for the hospital **(*n* = 1127)**	Supportive services
Sockalingam et al. 2020 [42*], Canada	Health care professionals (HCPs)** (*n* = 426)**	ECHO-CWC was scheduled as two 1-h sessions per week and included the following components: introductions, **a mindfulness exercise, COVID-19 information question and answer, a library update on resources, a didactic presentation based on the curriculum topic, case-based discussion to illustrate stress management skills, and a closing section based on health humanities education** (eg, poem or art) to stimulate reflection and community connectedness.
Teall et al. 2021 [33*], USA	Nurses **(*n* = 104)**	Areas in the program that were emphasized included (1) **personalized support for wellness; (2) prioritizing physical activity, healthy eating, sleep, and stress management; and (3) establishment of strength-based, sustainable solutions to improve well-being.**
**Qualitative interview studies (*n* = 3)**		
Blake et al. 2021 [43*], UK	Employees **(*n* = 24)** from an acute hospital trust in the UK	**Supported wellbeing centres** were set up in UK hospital trusts as an early intervention aimed at mitigating the psychological impact of COVID-19 on healthcare workers. These provided **high quality rest spaces with peer-to-peer psychological support from wellbeing buddies** trained in psychological first aid.
San Juan et al. 2020 [44*], UK	Front-line healthcare workers** (*n* = 33)**	**Well-being guidelines**
Yoon et al. 2021 [45*], Singapore	Frontline workers **(*n* = 42)**	**Mobile Health Apps** to Support Psychosocial Well-being Among Frontline Health Care Workers Involved in the COVID-19 Pandemic
**Reviews (*n* = 4)**		
Ardekani et al. 2021 [23*], Iran	Undergraduate medical students (***n*** = **10** studies)	Two major themes were identified: **(a) academic support and (b) mental health support**. All of the included studies utilized **online methods** whether for transitioning from previous support systems or developing novel approach
Buselli et al. 2021 [24*], Italy	Healthcare workers outbreak (***n*** = **7** studies)	Only few countries have published specific psychological support intervention protocols for HCWs. All programs were developed in university associated hospitals and highlighted the importance of multidisciplinary collaboration. All of them had as their purpose to manage the psychosocial challenges to HCW’s during the pandemic in order to prevent mental health problems.
Drissi et al. 2021 [25*], United Arab Emirates, Spain, Morocco	Healthcare workers (***n*** = **11** studies)	The identified e-mental health interventions consisted of **social media platforms, e-learning content, online resources and mobile applications**
Hooper et al. 2021 [26*], Australia	Healthcare workers and security forces (***n*** = **12** studies)	Early psychological interventions for individuals trained to provide services in emergency or disaster settings

Participants were HCWs (nurses, physicians, and other hospital staff) mostly working on the front-line and in direct contact with infected patients. One study concerned undergraduate medical students. Number of participants in studies ranged from 6 to 1127, and number of studies in the systematic reviews ranged from 7 to 24. All studies were published since the beginning of the COVID-19 pandemic (2020–2022), except for one study that was published in 2006 after SARS pandemic. Studies concerning MERS pandemic was not included (Table 2).

Study designs included six intervention studies including RCTs (*n* = 4), and quasi-experimental studies (*n* = 2). Studies were also cross-sectional (*n* = 6), qualitative interview (*n* = 3), and systematic reviews (*n* = 4) (Online only material 6).

### Emotional support programs and interventions for healthcare professionals, and students following pandemics

Most of the interventions (64.3%, 9/14) involved online or digital solutions (Table 2, Online only material 6), such as video interventions, presentations, or video-based debriefing program [34*, 40*], smartphone or a mobile phone-based applications [37*, 38*, 45*], a psychological assistance hotline [32*], and a virtual Balint-based approach using Zoom application [36*]. In person face-to-face interventions were also used.

The content of the interventions included efforts to increase treatment-seeking by HCWs during the COVID-19 pandemic [34*], expressive writing (EW) [31*, 37*], adaptive emotion regulation activities or positive emotion activities [37*], emotional skills, healthy lifestyle behavior, burnout, and social support [38*], and mindfulness practices [42*].

The content of the support programmes included ‘Supported Wellbeing Centres’ which were relaxing spaces with comfortable seating, relaxing music, low-level lighting, plants, and aromatherapy [39*, 43*], in-service training, including manpower allocation, gathering sufficient protective equipment, and establishment of a mental health team [35*], and a brief emotional awareness enhancing module with a Balint-based approach [36*]. Some programs included personalized support for wellness prioritizing physical activity, healthy eating, sleep, and stress management and establishment of strength-based, sustainable solutions to improve well-being [33*]. Among interventions, 35.7% were self-administered, 42.9% were provided under professionals’ responsibility and 21.4% had both characteristics (Table 3).

**Table 3:** Nature (digital / self-administered) of interventions (reviews not included)

	**Digital / on-line**	**Self-administered**
Amsalem et al. 2022 [34*]	Yes, video	Yes
Blake et al. 2020 [39*], 2021 [43*]	No	Yes
Chen et al. 2006 [35*]	No	No
Chochol et al. 2021 [36*]	Yes, virtual Balint-based approach (Zoom)	No
Coifman et al. 2021 [37*]	Yes, smartphone app	Yes
Fiol-DeRoque et al. 2021 [38*]	Yes, mobile phone-based app	Yes
Geoffroy et al. 2020 [32*]	Yes, hotline	No
Monette et al. 2020 [40*]	Yes, video debriefing (Zoom)	No
Petrella et al. 2021 [41*]	Yes	Both
Procaccia et al. 2021 [31*]	No	Yes
Sockalingam et al. 2020 [42*]	Yes, tele-education	No
Teall et al. 2021 [33*]	No	No
Vera San Juan et al. 2020 [44*]	No	Both
Yoon et al. 2021 [45*]	Yes, mobile apps	Both

Among reviews, support programs consisted of 1) academic support or 2) mental health support [23*]. Many were early psychological interventions [26*], to manage the psychosocial challenges to HCW’s during the pandemic [24*, 25*]. Online or e-mental health solutions (social media platforms, e-learning content, online resources, and mobile applications) were common [23*, 25*].

### The impact of emotional support programs and interventions

#### Intervention studies (*n* = 6)

In an RCT, Amsalam et al. [34*] studied the efficacy of brief video-based intervention. This resulted greater increases in treatment-seeking intentions vs control, particularly among participants in the repeat-video group. Exploratory analysis revealed that in both video groups, the effect was greater among nurses than non-nurses. The study by Coifman et al. [37*] tested the efficacy of an online ambulatory intervention to support psychological health and well-being for medical personnel. They found a 13% increase in positive emotions and decrease in negative emotion by 44% with good compliance and acceptability ratings. Procaccia et al. [31*] conducted an RCT to investigate the efficacy of an EW intervention. Participants who received the EW intervention showed greater improvements in PTSD, depression, and global psychopathology symptoms. Contradictory findings were obtained from an RCT conducted by Fiol-DeRoque et al. [38*] of the effectiveness of a psychoeducational, mindfulness-based mHealth intervention to reduce mental health problems in health care workers during the COVID-19 pandemic. After 2 weeks, there were no significant differences in outcomes between the groups. However, the usability and acceptability of the intervention were still high.

In a SARS program developed by Chen et al. [35*], the anxiety and depression and sleep quality of nursing staff started to improve 2 weeks after the initiation of the intervention, which was also experienced as feasible and rated acceptable. Similarly, in a pilot study by Chochol et al. [36*] trainees found a reduction in burnout and improvements in enthusiasm, and empathy with colleagues, and connectedness with colleagues and patients at work, as well as improvements in happiness and valued contributions at work. This intervention was also found to be feasible and acceptable.

#### Cross-sectional studies (*n* = 6)

In cross-sectional study results describing views and experiences of participants, interventions were mostly seen as positive. Blake et al. [39*] asked about the usage and views of Supported Wellbeing Centres and found that wellbeing was higher in those that accessed a wellbeing centre. The centres were described as very supportive spaces away from the stress of the hospital. Geoffroy et al. [32*], implemented a psychological assistance hotline team. They found a need for psychological support system and that this kind psychological support system could be easily duplicated and seemed to benefit all HCWs.

A video-based debriefing program to support emergency clinician well-being by Monette et al. [40*] was found to be an acceptable and useful approach to support emotional well-being. Based on work by Teall et al. [33*] wellness support helped nurses engage in self-care and wellness, and to improve their mental and physical health. Petrella et al. [41*] studied the use of supportive services made available during the acute phase of the COVID-19 pandemic and found that although HCWs were aware of supportive services, uptake varied. However, the majority of staff used at least one service (most common was free food, followed by donations and care packages and an off-site respite centre) and rated it as helpful. The study by Sockalingam et al. [42*] of Project Extension for Community Healthcare Outcomes (ECHO) Coping with COVID (ECHO-CWC) found that participants were highly satisfied with the programme and that it could be rapidly mobilized to address HCWs’ mental health needs during the COVID-19 pandemic.

#### Qualitative interview studies (*n* = 3)

Blake et al. [43*] interviewed study participants about ‘Supported Wellbeing Centres’. They found that these were viewed as critical for the wellbeing of hospital employees during the COVID-19. They also found that this kind of initiatives requires managerial advocacy and to address job-related barriers to work breaks and accessing staff wellbeing resources. Vera San Juan et al. [44*] interviewed HCWs on the applicability of well-being guidelines in practice. They found that guidelines placed greater emphasis on individual mental health and psychological support, whereas healthcare workers placed greater emphasis on structural conditions at work, responsibilities outside the hospital and the support of the community. The well-being support interventions proposed in the guidelines did not always respond to the lived experience of staff, as some reported not being able to participate in these interventions because of under-staffing, exhaustion or clashing schedules. Yoon et al. [45*] interviewed frontline workers’ experience of psychological wellness programs and their perceptions of the usefulness of mHealth apps and found that personalized tailoring was valued, while frequent coaching and messages were seen as a distraction, and only a few participants appreciated a gamification function. Frontline workers described a need for ongoing social support and peer support community, but there were concerns about virtual peer interactions.

#### Reviews (*n* = 4)

Buselli et al. [24*] studied programs to manage the psychosocial challenges to HCW’s during the pandemic. They found that whether one program offers distinct benefit compared to the others cannot be known given the heterogeneity of the protocols and the lack of a rigorous protocol and clinical outcomes. Similarly, Drissi et al. [25*] studied e-mental health interventions and found less than third of studies included empirical evaluation of the reported interventions, and about half listed challenges and limitations related to the adoption of the reported interventions. Feedback on the identified interventions was mostly positive, but there was a lack of empirical evaluation of these interventions. Another gap in the research evidence was the lack of RCTs, to provide rigorous evidence. They further noted that facilitators and barriers to the implementation of these interventions should be identified. Ardekani et al. [23*] found that students seem to be receptive to these new systems. They also found that most studies merely described only very positive effects of the program rather than providing a more comprehensive evaluation of these systems. Hooper et al. [26*] studied early psychological programmes aiming to prevent or reduce mental health symptoms and found that despite the limited evidence, psychological first aid, eye movement desensitisation and reprocessing, and trauma risk management showed effectiveness with frontline workers across a variety of disaster situations.

## Discussion

This systematic review synthesized the characteristics of emotional support programs and interventions targeted at healthcare workers and students during and after COVID-19 and other SARS-CoV pandemics, and described the effectiveness and experiences of these programs. These programs have focused on professionals in the frontline of care for infected patients. Most of the interventions were found to be effective and were experienced to be feasible, useful, helpful, and acceptable. Relatively few criticisms were presented, and on systematic review found a positive bias in reporting in most reports [23*]. It is possible that HCW recipients of the interventions were grateful for any intervention to support their mental health and wellbeing. However, some studies have highlighted that some workers did not recognize any problems and refused any psychological help [22, 46]. It is important to support HCW staff in different ways and to prevent them to becoming second victims that are, based on definition “Any health care worker, directly or indirectly involved in an unanticipated adverse patient event, unintentional healthcare error, or patient injury and who becomes victimized in the sense that they are also negatively impacted” [47].

The main focus of the interventions identified in this study was on providing emotional support at a critical moment of the COVID-19 pandemic, seeking to reinforce the work capacity of frontline professionals. The same is true for the intervention reviewed in the case of SARS. This probably justifies that not enough evidence has been provided on the effectiveness of these interventions, although there was a positive experience of the recipients of the programs. For the same reason, the urgency of providing some type of support helps explain the biases noted in the studies. The motivation for implementation of these support interventions has been the urgent need to act so that the entire healthcare workforce has felt supported. Three strategic principles have been pointed out: providing leadership focused on resilience, structuring crisis communications to provide information and empowerment, and creating a continuum of staff support within the organization [48].

The majority of the interventions studied were online interventions, likely due to pandemic imposed limits to in-person contacts. A benefit of this kind of interventions is easy accessibility. On the other hand, these does not capture the need for support in the workplace. For example, in the UK, hubs were developed during the COVID-19 pandemic, a number of which provided in situ ‘wobble rooms’ featuring, face-to-face counselling and food and drinks as an opportunity for informal support and conversations (e.g. [39*, 43*]). Several participants also noted anecdotally that any intervention requiring removal of PPE would create a major barrier to access. This suggests that both online and face-to-face interventions, have their benefits and may be useful in different situations and for different people.

Interventions using a psychoeducational approach, mindfulness or spaces for rest and recovery of professionals were the most common. However, there has been little standardization of the contents and design of the interventions. In addition, study designs have not permitted the evaluation of which intervention components work and which do not. It has been found that the interventions that have encouraged more participative leadership styles, organizational culture that facilitates interdisciplinary teamwork and the feedback of information provided by middle managers and supervisors have been positive in minimizing the negative impacts of working in the pandemic [49]. This suggests that development and deployment of resources to support the health care workforce and address new outbreaks should take into account differences in organizational culture (e.g., psychological safety), and availability of human and financial resources in different countries [49].

Pollock et al. [50] found that an important barrier to utilization of interventions was a lack of awareness that they existed by frontline workers. Other barriers included a lack of equipment to deliver the interventions to large numbers of staff members, and lack of time needed to receive the intervention. Additionally, frequent gaps identified in the COVID-19 outbreak were in organization, human and materials resources, training, information, and individual and team psychological responses. Also, the institutional role in assessing the effectiveness of mental health interventions at the organizational level for healthcare workers during or after a public health emergency, such as pandemics, are limited if not lacking [51]. On the other hand, facilitators of implementation were interventions that could be adapted for local needs; effective communication about the intervention, both formally and socially; and a positive, safe, and supportive learning environments for frontline workers [50].

### Limitations

A limitation of this study is that it presents only a narrative synthesis of study results. However, the data could not be pooled due to excessive heterogeneity among studies and the lack of common variables of interest. Similar results were reported by Buselli et al. [24*]. In addition, the majority of interventions lacked robust empirical evaluation, as noted previously [25*, 47]. Of the included 19 studies, only four were RCTs [27]. It is also difficult to demonstrate the comparative effectiveness interventions and their components to support HCWs during pandemics. The methodological quality of many included studies was rated as poor because of the high risk of bias in most indicators. Thus, their findings should be interpreted with caution. Finally, HCWs were mostly treated in aggregate, thus we were not able to describe what kind of support works better for subgroups of workers, such as physicians or nurses.

A strength of this study is the mixed methods nature of the design, which allowed presentation of data on experiences with these interventions as well as their effectiveness. Information on whether an intervention is feasible, meaningful, and appropriate to HCWs increases the usefulness of the findings for decision making and practice [27]. The inclusion of systematic reviews to this review also strengthened the findings.

## Conclusion

A number of promising interventions have been developed in the last few years to support health workers, many of them motivated by pandemic related stresses. However, based on the current literature it is difficult to conclude whether one program offers distinct benefits compared to the others. More evaluations and more rigorous designs are required in the future to determine the comparative effectiveness of emotional support interventions for health workers.

## Supplementary Information

**Additional file 1.** Quality evaluation of selected RCT studies (*n* = 4).

**Additional file 2.** Quality evaluation of selected Quasi-experimental studies (*n* = 2).

**Additional file 3.** Quality evaluation of selected Cross-sectional studies (*n* = 6).

**Additional file 4.** Quality evaluation of selected Qualitative studies (*n* = 3).

**Additional file 5.** Quality evaluation of selected Systematic Reviews (*n* = 4).

**Additional file 6.** Impact of emotional support programs and interventions (*n* = 19) for healthcare professionals, and students following pandemics.

## Data Availability

The datasets used and/or analysed during the current study available from the corresponding author on reasonable request.

## Abbreviations

CINAHL: Cumulative Index to Nursing and Allied Health Literature
COVID-19: Coronavirus disease 2019
COVIDENCE: Systematic review management system
ECHO: Extension for Community Healthcare Outcomes
ECHO-CWC: Extension for Community Healthcare Outcomes coping with COVID
HCW: Healthcare workers
JBI: Joanna Briggs Institute
Medline: National Library of Medicine’s (NLM) premier bibliographic database
PRISMA: The Preferred Reporting Items for Systematic Reviews and Meta-Analyses
PROSPERO: Prospective Register of Systematic Reviews
PsycINFO: The premier abstracting and indexing database covering the behavioral and social sciences from the authority in psychology
RCT: Randomized controlled trial
SARS-CoV: The severe acute respiratory syndrome coronavirus
SCOPUS: Elsevier’s abstract and citation database
WHO: The World Health Organization
