# Supplementary material for: The impact of emotional support on healthcare workers and students coping with COVID-19, and other SARS-CoV pandemics – a mixed-methods systematic review
Source: BMC Health Serv Res. 2023 Jul 13;23:751. doi: 10.1186/s12913-023-09744-6 (PMC10339499; doi:10.1186/s12913-023-09744-6)
Supplement: Supplementary file 1 — Additional file 1. Quality evaluation of selected RCT studies (n = 4). [file 12913_2023_9744_MOESM1_ESM.pdf]

Online only material 1. Quality evaluation of selected RCT studies (n=4)

	Randomization	Concealment of allocation	Treatment groups similar at the baseline	Participants were blinded	Treatment deliverers were blinded	Outcomes assessors were blinded	Study groups treated identically, except for intervention	Follow-up complete	Intention-to-treat analysis	Outcomes measured in the same way in all groups	Outcomes measured in a reliable way	Appropriate statistical analysis	Trial design appropriate for the topic	The percentage of compliance with the quality criteria
Amsalen 2022	√	√	√	?	x	?	√	√	√	√	√	√	√	69%
Coifman 2021	√	√	√	√	x	?	√	?	√	√	x	√	√	69%
Fiol-DeRoque 2021	√	√	√	√	x	√	√	√	√	√	√	√	√	92%
Procaccia 2021	?	?	?	x	x	x	?	x	√	√	x	√	√	31%

√: Yes; x: No; ?: Unclear; NA: Not applicable
