# Supplementary material for: The impact of emotional support on healthcare workers and students coping with COVID-19, and other SARS-CoV pandemics – a mixed-methods systematic review
Source: BMC Health Serv Res. 2023 Jul 13;23:751. doi: 10.1186/s12913-023-09744-6 (PMC10339499; doi:10.1186/s12913-023-09744-6)
Supplement: Supplementary file 2 — Additional file 2. Quality evaluation of selected Quasi-experimental studies (n = 2). [file 12913_2023_9744_MOESM2_ESM.pdf]

Online only material 2. Quality evaluation of selected Quasi-experimental studies (n=2)

	Cause and effect variables	Participants from the compared groups	Other exposures/treatments occurring in the same time	Control group	Multiple measurements of the outcomes	Complete follow-up	Outcomes measured in the same way	Outcomes measured in a reliable way	Statistical analysis	The percentage of compliance with the quality criteria
Chochol 2021	√	√	?	x	√	√	√	√	x	67%
Chen 2006	√	√	√	x	√	√	√	√	√	89%

√: Yes; x: No; ?: Unclear; NA: Not applicable
