# Supplementary material for: The impact of emotional support on healthcare workers and students coping with COVID-19, and other SARS-CoV pandemics – a mixed-methods systematic review
Source: BMC Health Serv Res. 2023 Jul 13;23:751. doi: 10.1186/s12913-023-09744-6 (PMC10339499; doi:10.1186/s12913-023-09744-6)
Supplement: Supplementary file 3 — Additional file 3. Quality evaluation of selected Cross-sectional studies (n = 6). [file 12913_2023_9744_MOESM3_ESM.pdf]

Online only material 3. Quality evaluation of selected Cross-sectional studies (n=6)

	Clear inclusion criteria	Detailed description of subjects and setting	Exposure measured in a valid and reliable way	Standard criteria used for measurement of the condition	Confounding factors identified	Strategies to deal with confounding factors	Outcomes measured in a valid and reliable way	Appropriate statistical analysis	The percentage of compliance with the quality criteria
Blake 2020	√	√	?	x	x	x	√	√	50%
Geoffroy 2020	?	√	?	NA	NA	NA	NA	√	25%
Monette 2020	√	√	√	√	x	x	x	√	62%
Petrella	√	√	√	√	x	x	√	√	75%
Sockalingam	√	√	√	√	x	x	x	√	62%
Teall	√	x	x	NA	NA	NA	x	x	12%

√: Yes; x: No; ?: Unclear; NA: Not aplicable
