# Supplementary material for: The impact of emotional support on healthcare workers and students coping with COVID-19, and other SARS-CoV pandemics – a mixed-methods systematic review
Source: BMC Health Serv Res. 2023 Jul 13;23:751. doi: 10.1186/s12913-023-09744-6 (PMC10339499; doi:10.1186/s12913-023-09744-6)
Supplement: Supplementary file 4 — Additional file 4. Quality evaluation of selected Qualitative studies (n = 3). [file 12913_2023_9744_MOESM4_ESM.pdf]

Online only material 4. Quality evaluation of selected Qualitative studies (n=3)

	Congruity between philosophical perspective and methodology	Congruity between methodology and the objectives	Congruity between methodology and methods used to collect data	Congruity between methodology and representation and analysis of data	Congruity between methodology and the interpretation	Statement locating the researcher culturally or theoretically	Influence of the researcher on the research	Participants adequately represented	Ethical research	Conclusions report flow from the data analysis	The percentage of compliance with the quality criteria
Blake 2021	√	√	√	√	√	x	x	√	√	?	70%
Vera San Juan 2020	√	√	√	√	√	x	x	√	√	√	80%
Yoon	√	√	√	√	√	x	x	√	√	?	70%

√: Yes; x: No; ?: Unclear; NA: Not applicable
