# Supplementary material for: The impact of emotional support on healthcare workers and students coping with COVID-19, and other SARS-CoV pandemics – a mixed-methods systematic review
Source: BMC Health Serv Res. 2023 Jul 13;23:751. doi: 10.1186/s12913-023-09744-6 (PMC10339499; doi:10.1186/s12913-023-09744-6)
Supplement: Supplementary file 5 — Additional file 5. Quality evaluation of selected Systematic Reviews (n = 4). [file 12913_2023_9744_MOESM5_ESM.pdf]

Online only material 5. Quality evaluation of selected Systematic Reviews (n=4)

	Clear and explicit review question	Appropriate inclusion criteria	Appropriate search strategy	Adequate information sources	Appropriate critical appraisal	Critical appraisal by two or more reviewers	Methods to minimize errors in data extraction	Appropriate methods used to combine studies	Publication bias assessment	Recommendations for policy and/or practice supported by the reported data	Appropriate specific directives for new research	The percentage of compliance with the quality criteria
Ardekani	?	?	√	√	√	√	√	?	NA	√	√	64%
Buselli	√	√	?	√	?	x	x	NA	√	NA	√	45%
Drissi	√	√	x	√	x	x	x	NA	x	x	x	27%
Hooper	√	√	?	√	√	√	√	√	√	√	√	91%

√: Yes; x: No; ?: Unclear; NA: Not applicable
