# Supplementary material for: The impact of emotional support on healthcare workers and students coping with COVID-19, and other SARS-CoV pandemics – a mixed-methods systematic review
Source: BMC Health Serv Res. 2023 Jul 13;23:751. doi: 10.1186/s12913-023-09744-6 (PMC10339499; doi:10.1186/s12913-023-09744-6)
Supplement: Supplementary file 6 — Additional file 6. Impact of emotional support programs and interventions (n = 19) for healthcare professionals, and students following pandemics. [file 12913_2023_9744_MOESM6_ESM.pdf]

Online only material 6. Impact of emotional support programs and interventions (n=19) for healthcare professionals, and students following pandemics

**Study & design**	**Aim**	**Programme / intervention**	**Impact of emotional support programmes and interventions**
**Intervention studies (n=6)**			
Amsalem et al. 2022, USA<br><br>RCT	To evaluate the **efficacy** of a brief, social contact-based video intervention in increasing treatment-seeking intentions among healthcare workers	The 3 min **video** to increase treatment-seeking by healthcare workers during the COVID-19 pandemic	**Outcome**: <u>Treatment-seeking intentions</u><br>The brief video-based intervention yielded greater increases in treatment-seeking intentions than the control condition, particularly among participants in the repeat-video group. Exploratory analysis revealed that in both video groups, greater effect among nurses than non-nurses were found.
Chen et al. 2006, Taiwan<br><br>Quasi-experimental	To determine the anxiety, depression, and sleep quality a SARS nursing staff **experienced before and after** a SARS prevention program.	**Face-to-face** in-service training, manpower allocation, gathering sufficient protective equipment, and establishment of a mental health team	**Outcomes**: <u>self-rating anxiety, self-rating depression, sleep quality</u>.<br>Nursing staff's anxiety and depression along with sleep quality started to improve 2 weeks after the initiation of SARS prevention controls.
Chochol et al. 2021, USA<br><br>Quasi-experimental	To enhance professional fulfillment and support while decreasing risk and prevalence of burnout in Child and Adolescent Psychiatry (CAP) trainees	A brief emotional awareness enhancing module with a **virtual** Balint-based approach using Zoom application	**Outcome**: <u>Well-being Index (well-being and likelihood of burnout), Stanford Professional Fulfilment Index (professional fulfillment and presence of burnout), survey questions to assess trainees' sense of professional support and screen for burnout</u>.<br>Trainees found the curriculum feasible and useful. Surveys showed a reduction in burnout from three to zero participants (p= 0.03) and specific improvements in enthusiasm (p= 0.013), empathy with colleagues (p= 0.093), and connectedness with colleagues (p= 0.007) and patients (p= 0.042) at work. There were also improvements in happiness (p= 0.042) and valued contributions at work (p=0.004).
Coifman et al. 2021, USA<br><br>RCT	To test the **efficacy** of a brief and novel online ambulatory intervention aimed at supporting psychological health and well-being for medical personnel and first responders	Each daily intervention with **smartphone** application included expressive writing, adaptive emotion regulation activity and positive emotion activities, lasting 3–6 min a day.	**Outcome**: <u>intensity of specific negative (disgust, anger, sadness, fear, distress) and positive (happiness, amusement, affection, contentment, relief) emotions</u>.<br>The results indicated a 13% increase in positive emotion, t(25)=2.01, p=0.056; and decrease in negative emotion by 44%, t(25)=−4.00, p=0.001. However, there was a clear advantage for individuals in the high-dose condition as daily boosts in positive emotion were significantly greater (an additional 9.4%) B=0.47, p=0.018. Overall, compliance was good. Acceptability ratings were good for those who completed the follow-up assessment.

Fiol-DeRoque et al. 2021, Spain<br><br>RCT	To evaluate the **effectiveness** of a psychoeducational, mindfulness-based mHealth intervention to reduce mental health problems in HCWs during the COVID-19 pandemic.	PsyCovidApp intervention (a mobile phone-based app targeting emotional skills, healthy lifestyle behavior, burnout, and social support)	**Outcome**: <u>an overall index of depression, anxiety, and stress</u>.<br>No significant differences were observed between the groups at 2 weeks in the primary outcome (standardized mean difference -0.04; 95% CI -0.11 to 0.04; P=.15) or in the other outcomes. The mean usability score of PsyCovidApp was high (87.21/100, SD 12.65). After the trial, 208/221 participants in the intervention group (94.1%) asked to regain access to PsyCovidApp, indicating high acceptability.
Procaccia et al. 2021, Italy<br><br>RCT	To investigate the **efficacy** of an expressive writing intervention, and to analyze if outcomes of EW vary in function of individual differences	Expressive writing (EW) intervention.	**Outcome**: <u>ptsd, depression and global psychopathology's symptoms (somatization, obsessive-compulsive disorder, interpersonal sensitivity, depression, anxiety, hostility, phobic anxiety, paranoid ideation, psychoticism, and sleep disturbances), perceived social support (significant other, family, and friend), and resilience</u>.<br>EW intervention showed higher improvements in ptsd, depression, and global psychopathology symptoms. Improvements in EW group varied in function of age, gender, marital status, and baseline values: young, men, married participants and those who had higher baseline scores showed a higher reduction of psychological distress symptoms while women, single and those who had lower baseline value showed increased social support, and resilience
**Cross-sectional studies (n=6)**			
Blake et al. 2020, UK	To determine Supported Wellbeing Centres **usage** and gather insight into employee wellbeing and the **views** of employees towards this provision.	Supported Wellbeing Centres	**Outcome**: <u>working in COVID-19 high or low risk area, centre access, buddy contact, and perceived benefits and barriers.</u><br>Facilities were highly valued, but the service model was resource intensive. Wellbeing was higher in those that accessed a wellbeing centre. The centres were described as 'very tranquil', 'a supportive place for staff' and 'a great space to come and sit away from the stress of the hospital'. Buddies were described as 'very friendly', 'approachable' and 'easy to talk to'.
Geoffroy et al., 2020, France	To rapidly design and implement a psychological support system for all hospital workers in Paris during the Covid-19 outbreak.	The psychological assistance hotline team	**Outcome**: e.g. <u>reasons for hotline calls</u>.<br>Reasons for calling were anxiety symptoms (n=73, 49%), request for hotline information (n=31, 20.8%), worries about Covid-19 (n=23, 15.44%), exhaustion (n=17, 11.41%), trauma reactivation (n=10, 6.11%), insomnia (n=9, 6.0%), anger (n=8, 5.37%), depressive (n=6, 4.02%), and psychotic symptoms (n=3, 2.01%). This psychological support system can be easily duplicated and seems to benefit all hospital professions.

Monette et al. 2020, USA	To describe the program and its **acceptability** and **initial impact** on emergency clinicians providing care	A video-based (Zoom) debriefing program to support emergency clinician well-being	**Outcome**: experience with the program<br>Emergency clinicians participating in a video-based debriefing program during the coronavirus pandemic found it to be an acceptable and useful approach to support emotional well-being. The program provided participants with a platform to support each other and maintain a sense of community and connection.
Petrella et al. 2021, UK	To assess HCW psychological welfare and their **use of** supportive services	Supportive services during the acute phase of the COVID-19 pandemic	**Outcomes**: well-being (self-rated health, moral distress exposure, symptoms of burnout and psychological distress) and use of available supportive services (awareness of, use and perceived helpfulness).<br>HCWs experienced high levels of psychological distress. Although HCWs were aware of supportive services, uptake varied. Majority of staff used at least one service and rated it as helpful.
Sockalingam et al. 2020, Canada	To describe the psychological needs of HCPs during COVID-19 and the **implementation of Project Extension** for Community Healthcare Outcomes (ECHO) Coping with COVID (ECHO-CWC) to help HCPs manage COVID-19 distress	ECHO-CWC included e.g a mindfulness, COVID-19 information, a didactic presentation, case-based discussions, and a closing section based on health humanities education	**Outcomes**: participants' needs (participants' perceived risk of COVID-19 and self-efficacy), participant engagement and session satisfaction scores.<br>Most participants reported feeling increased stress at work (84.5%), fear of infecting others (75.2%), and fear of falling ill (70.5%) from COVID-19, yet most participants accepted the risk associated with work during this time (59.7%). Participants were highly satisfied with the initial five sessions (mean = 4.26). Using an iterative curriculum design approach and existing implementation frameworks, the ECHO tele-education model can be rapidly mobilized to address HCPs' mental health needs during the COVID-19 pandemic
Teall et al. 2021, USA	To enhance the health and well-being of nurses on the front lines during the COVID-19	The wellness support program	**Outcome**: to review wellness support implementation and the impact of uncertainty, challenges of behavior change, coping strategies for stress and burnout, use of skillful questioning, and the importance of self-care, self-efficacy, and resilience.<br>98% of nurses shared that the wellness support program helped them engage in self-care and wellness, and 94.7% agreed or strongly agreed that The Wellness Partner Program helped them improve their mental and physical health.
**Qualitative interview studies (n=3)**			
Blake et al. 2021, UK	To explore the **views of wellbeing centre** visitors and operational staff towards this COVID-19 workforce wellbeing provision.	Supported wellbeing centres	**Outcome**: the emotional impact of the pandemic on participants and participants' views towards the wellbeing centres and wellbeing buddies.<br>Wellbeing centres were viewed as critical for the wellbeing of HCWs during the COVID-19. Wellbeing initiatives require managerial advocacy. Job-related barriers were work breaks and accessing staff wellbeing provisions. High quality rest spaces and access to peer-to-peer

			support were seen to benefit individuals, teams, organisations and care quality.
Vera San Juan et al. 2020, UK	To assess the **applicability of well-being guidelines** in practice, identify unaddressed healthcare workers' needs and provide recommendations for supporting front-line staff.	Well-being guidelines	**Outcome**: the applicability of well-being guidelines in practice, identify unaddressed healthcare workers' needs and provide recommendations for supporting staff.<br>The guidelines placed greater emphasis on individual mental health and psychological support, whereas HCWs placed greater emphasis on structural conditions at work, responsibilities outside the hospital and the invaluable support of the community. The well-being support interventions proposed in the guidelines did not always respond to the lived experience of staff, as some reported not being able to participate in these interventions because of under-staffing, exhaustion or clashing schedules
Yoon et al. 2021, Singapore	To explore frontline workers' **experience of conventional psychological wellness programs** and their perceptions of the **usefulness** of mHealth apps and features for promoting well-being, as well as to identify factors that could influence uptake and retention of an mHealth-based wellness program.	Mobile Health Apps to Support Psychosocial Well-being	**Outcome**: perceptions of the existing wellness program available for frontline workers, perceived usefulness of mHealth apps for mental well-being, features that might be valuable for improving wellness, and factors affecting adoption of mHealth apps for wellness.<br>A personalized goal-setting feature (ie, tailoring) and in-app resources were generally valued, while frequent coaching and messages (ie, framing) were seen as a distraction. The majority of participants desired a built-in chat function with a counselor (ie, guidance) for reasons of accessibility and protection of privacy. Very few participants appreciated a gamification function. Frontline workers commonly reported the need for ongoing social support and desired access to an in-app peer support community (ie, social influence). There were, however, concerns regarding potential risks from virtual peer interactions. Intrinsic motivational factors, mHealth app technicality, and tangible rewards were identified as critical for uptake and retention.
**Reviews (n=4)**			
Ardekani et al. 2021, Iran	To review the current literature regarding medical student support in response to the COVID-19	Studies included academic support or mental health support.	**Outcome**: student support system programs in any stage of undergraduate medical education and studies describing student support systems explicitly used in response to the COVID-19 pandemic.<br>Students and faculty members seemed to be receptive to these new systems. Despite indicating outstanding program outcomes, most studies merely described the positive effects of the program rather than providing a precise evaluation
Buselli et al. 2021, Italy	To ascertain the interventions put in	Programs managed psychosocial	**Outcome**: the study reported a protocol of intervention to address mental health of HCWs during COVID-19 outbreak.

	place worldwide in reducing stress in HCWs during the COVID-19 outbreak.	challenges to HCW's in order to prevent mental health problems.	Whether one program offers distinct benefit compared to the others cannot be known given the heterogeneity of the protocols and the lack of a rigorous protocol and clinical outcomes.
Drissi et al. 2021, United Arab Emirates, Spain, Morocco	To identify e-mental health interventions, reported in the literature, that are developed for HCWs during the COVID-19 pandemic	The e-mental health interventions	**Outcome**: e-mental health interventions for HCWs during the COVID-19 pandemic.<br>Only 27% of the studies included empirical evaluation of the reported interventions, 55% listed challenges and limitations related to the adoption of the reported interventions. Feedback on the identified interventions was positive, yet a lack of empirical evaluation was identified, especially regarding qualitative evidence
Hooper et al. 2021, Australia	To research on early psychological programmes that aim to prevent or reduce mental health symptoms and that have been tested in frontline responders	Psychological interventions for individuals trained to provide services in emergency or disaster settings	**Outcome**: early psychological intervention programmes tested in frontline responders; and a healthcare service evaluation framework that reviewed the suitability of each programme for widespread implementation across healthcare workers based on the criteria of effectiveness, content applicability and feasibility of delivery.<br>Although the evidence base is limited, psychological first aid, eye movement desensitisation and reprocessing, and trauma risk management showed effectiveness with frontline workers. Several interventions were identified as potentially suitable and useful for improving psychological functioning of healthcare workers across a variety of disaster situations.
HCW= Health care worker			
